# Transcriptome analysis reveals carbohydrate-mediated liver immune responses in *Epinephelus akaara*

**DOI:** 10.1038/s41598-017-18990-8

**Published:** 2018-01-12

**Authors:** Yunxia Yang, Tao Han, Jia Xiao, Xinyu Li, Jiteng Wang

**Affiliations:** 1 Department of Aquaculture, Zhejiang Ocean University, Zhoushan, China; 2 Department of Immunobiology, Jinan University, Guangzhou, China

## Abstract

As the cheapest energy source, carbohydrates are used in fish feeds to improve physical quality and reduce catabolism of proteins and lipids. The liver is the primary organ for metabolism and is also an important site of immune regulation. Here, we investigated the effect of different dietary carbohydrate levels on growth and health by evaluating the liver transcriptome of *Epinephelus akaara*. In this study, *E. akaara* juveniles were fed diets containing few (0% corn starch), moderate (18% corn starch), and high (30% corn starch) levels of dietary carbohydrate. After an 8-week feeding trial, *E. akaara* fed 30% dietary carbohydrates exhibited poor growth performance compared with those fed 0% and 18% dietary carbohydrates (*P* > 0.05). Genes related to the immune system, including IL8, TLR9, CXCR4, CCL4, and NFκB inhibitor alpha, were over-expressed in *E. akaara* fed the highest level of carbohydrate (30%). This general over-expression could indicate activation of inflammatory processes in the liver. The liver transcriptome data of *E. akaara* reported here indicate that high carbohydrate level of diet can lead to poor growth and inflammatory immune response in *E. akaara*.

## Introduction

Carbohydrates are the cheapest energy sources and the major compounds that make up organisms. Some studies have indicated that certain levels of carbohydrates can improve feed utilization and protein retention in rainbow trout (*Oncorhynchus mykiss*), Atlantic salmon (*Salmo salar*), European eel (*Anguilla anguilla*), Atlantic cod (*Gadus morhua*), and different carp species (*Catla catla*, *Labeo rohita*, *Cirrhinus mrigala*)^1–7^. If sufficient carbohydrate content is not provided in the diet, other nutrients such as proteins and lipid can be used for energy^8^; This could lead to an increase in cost and nutritional imbalances. In addition, post-prandial prolonged hyperglycemia occurs when a high level of carbohydrates is consumed^9–14^. An imbalance carbohydrates in the diet may make the fish under metabolic stress^15^ and have negative effects on nutrient retention, growth, metabolism, and health^16^. Thus, it is important to supply an appropriate level of carbohydrates in aqua-feeds.

The liver is the primary target organ for metabolism. Nutrients are absorbed into the body through the small intestine and then delivered to liver. The critical metabolic functions of the liver often eclipse its role as an important organ for immune regulation^17^, as the liver serves as a physical barrier responsible for filtering potentially harmful antigens, which may reach the body via the gastrointestinal tract. Castro *et al*.^18^ reported that the liver modulates the immune response following hemorrhagic septicemia virus (VHSV) infection in rainbow trout. Similarly, when inappropriate feed supplied, the liver also can function as an immunocompetent organ^19–22^. A growing body of research has focused on the relationship between carbohydrate supplementation and immune function. For example, carbohydrates can regulate the production of proinflammatory cytokines to enhance the endurance performance and attenuated stress hormone response^23^.

Carbohydrate utilization differs among fish species^16,24–26^. Herbivorous and omnivorous fish can utilize as much as 45% carbohydrate content in diet^27–29^, but carnivorous fish show significantly poorer growth when fed diets with 30% carbohydrate levels compared to those fed moderate carbohydrate levels (in general, ≤20%), such as Atlantic salmon^5^ and cobia (*Rachycentron canadum*)^30^. *Epinephelus akaara* (Temminck and Schlegel, 1842) is an important marine carnivorous fish with a high market value in Asia, To date, few nutrient requirements have been investigated in *E. akaara*, except for appropriate level of proteins^31^ and lipids^32^. To obtain the appropriate formulation of fish feed, it is an essential pre-requisite to understand the capacity of fish to utilize carbohydrates^8^. High-throughput sequencing can provide an unprecedented view of global gene expression and detailed molecular information on responses to nutrition metabolism. Therefore, we here focused on the liver transcriptome of *E. akaara* fed diets containing few (0%), moderate (18%), and high (30%) levels of carbohydrates using high-throughput sequencing to identify genes responsible for growth and immune system alterations.

## Results

### Growth performance and growth-related gene expression in the liver

The growth performance of *E. akaara* was measured by percentage weight gain (PWG). In this study, the final body weight (g/fish) in each group was 29.30 ± 3.32, 28.54 ± 2.87, and 24.02 ± 0.92, respectively; the PWG (%) were 276.16 ± 41.99, 266.26 ± 28.33, and 208.69 ± 12.68 in the C1, C2, and C3 groups, respectively. PWG generally decreased with increasing dietary carbohydrate (*P* > 0.05), and fish fed 30% carbohydrates exhibited the lowest PWG.

We evaluated genes belonging to the GH/IGF-system to show the relationship between growth and gene expression. Several components of the GH/IGF axis in each group exhibited different expression levels. The expression of GHR, IGF1, IGF2, IGFBP2, IGFBP3 (*P* < 0.05), IGFBP4, and IGFBP6 (*P* > 0.05) were decreased in the C2 and C3 groups compared to the C1 group, whereas IGFBP1 (*P* < 0.05), IGFBP5, and IGFBP7 (*P* > 0.05) were upregulated in the C3 group to levels comparable to the C1 and C2 groups (Table 1).

**Table 1:** The expression of growth-related genes in three groups.

Gene ID	name	C1	C2	C3
c56634_g3	GHR	11.8233	8.4724	6.9467
c49944_g2	IGFBP1	1.6992	4.3513	84.0755
c46871_g1	IGFBP2A	12.4657	11.8959	5.4010
c59085_g2	IGFBP2B	102.9990	97.3850	65.5878
c33486_g1	IGFBP4	0.9967	0.7571	0.7533
c44323_g3	IGFBP5	16.8376	20.6902	24.5923
c69225_g1	IGFBP6	0.6224	0.3081	0.1927
c49986_g1	IGFBP7	2.6987	3.8087	4.8402
c60956_g7	IGF1	78.6293	29.1721	17.9944
c59327_g1	IGF2	49.4954	6.9903	10.1116

### De novo assembly

There were 5,5912,920, 63,532,872, and 78,789,902 raw reads in the C1, C2, and C3 groups, respectively, generated by high-throughput sequencing of the cDNA library of the *E. akaara* liver. We cleaned the low-quality reads; the sequence of high quality rates were 87.13% (clean reads number: 48,717,774), 88.10% (55,971,730), 85.33% (67,230,310) in C1, C2, and C3 groups, respectively. There were 94% sequences up to the quality score of Q30, which describes quality score logarithmically linked to error probabilities (i.e., Q30 = 99.9%, chance correct base called). The sequencing results showed that these data were appropriate for analysis (Table 2). The ORF predictions came from the contigs assembled using Trinity. The contigs in each group were 83,451, 84,162, and 94,733, respectively. Subsequently, to annotate the sequences, blastp and blastx alignments (E-value < 10^−5^) with the NT, NR, gene, and string were used, and 27,327, 27,851, 31,234 contigs in each group featured a corresponding annotation (Table 2).

**Table 2:** Sequencing information of the *E. akaara* liver.

Samples	C1	C2	C3
Raw Reads Number	55,912,920	63,532,872	78,789,902
Clean Reads Number	48,717,774	55,971,730	67,230,310
Clean Reads Rate(%)	87.13	88.10	85.33
Low-quality Reads Number	4,827,778	5,897,502	7,384,488
Low-quality Reads Rate(%)	8.63	9.28	9.37
Ns Reads Number	3,910	4,422	5,330
Ns Reads Rate(%)	0.01	0.01	0.01
Adapter Polluted Reads Number	2,363,458	1,659,218	4,169,774
Adapter Polluted Reads Rate(%)	4.23	2.61	5.29
Raw Q30 Bases Rate(%)	89.93	90.04	88.98
Clean Q30 Bases Rate(%)	94.84	94.86	94.67
ORF counts	27327	27851	31234
ORF N50 (bp)	1011	1098	1074

### Functional annotation and analysis of differentially-expressed genes

**Figure 1:**
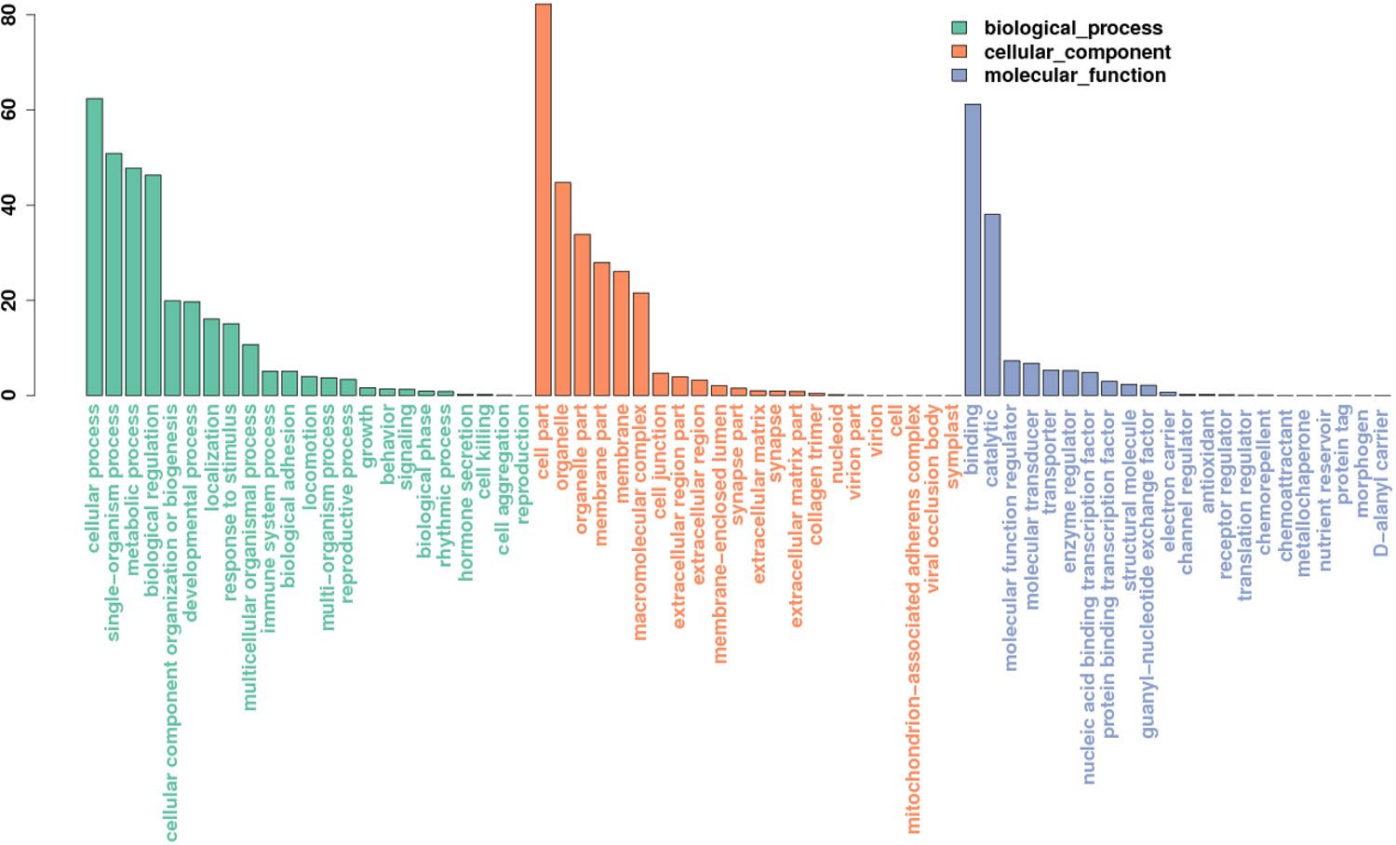
GO classification of assembled genes in the *E. akaara* liver transcriptome.

GO terms for the transcriptome were analyzed using Blast2GO, which provides information on “Biological Processes”, “Cellular Components” and “Molecular Function” for each contig (Fig. 1). In the “biological processes” categories, which features 23 subtypes, most corresponding genes were involved in cellular processes, single-organism processes, metabolic processes, and biological regulation. In addition, 22 subtypes were annotated with “cellular components”; most corresponding genes were involved in the cell, organelles, and parts of the membrane. In the “molecular function” category, which featured 22 subtypes, most corresponding genes were involved with binding and catalytic activity.

**Figure 2:**
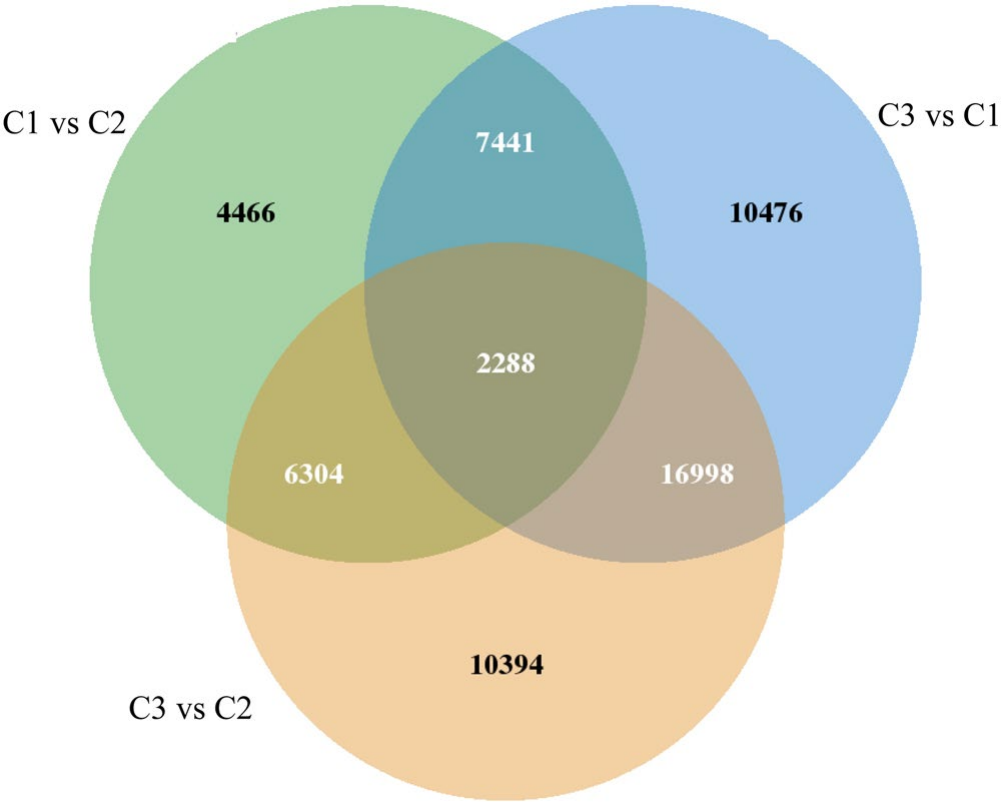
Differentially-expressed gene distribution. For example, 16,998 genes are differentially-expressed in C3 group compared with C1 and C2 groups.

The KEGG database was used to obtain more information to predict the unigene functions; 38,938 genes ware classified into 113 KEGG pathways. The KEGG pathway analysis was also used to identify genes observed to be differentially expressed in the C1-C2, C2-C3, and C1-C3 pair groups fed few (C1), moderate (C2), or high (C3) levels of carbohydrates. The numbers of differentially-expressed genes in each group (C1-C2, C2-C3, and C1-C3) were 20,499, 35,984, and 37,203, respectively (Fig. 2). There were 20,499 genes differentially expressed in the C2 group relative to the C1 group, 35,984 genes differently expressed in the C3 group relative to the C2 group, and 37,203 genes differently expressed in the C3 group relative to the C2 group. Compared with C1 and C2 group, the number of up-regulated gene in C3 group is no significant difference (*P* > 0.05).

**Figure 3:**
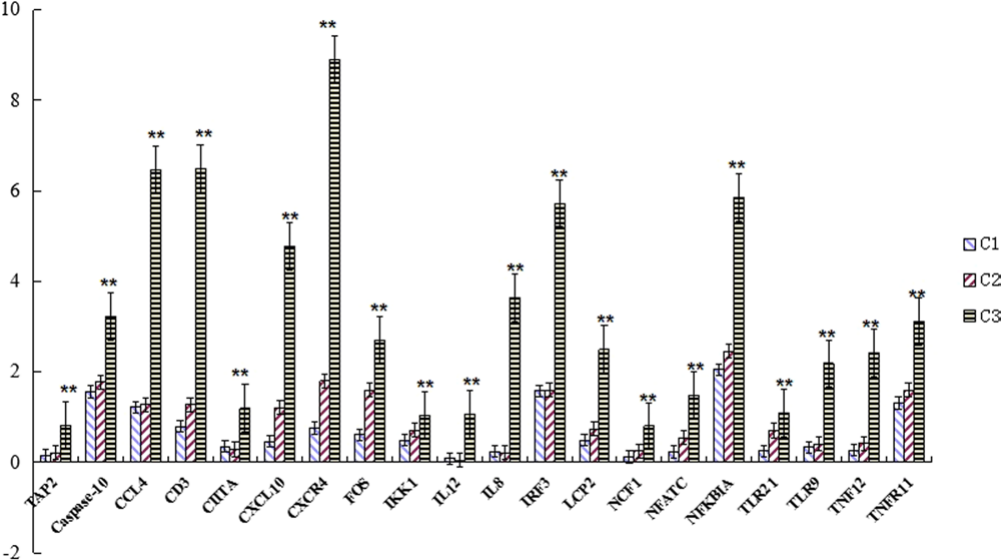
RT-qPCR confirmation of genes expressed at different carbohydrate level in C1, C2 and C3 groups. Bars represent mean ± standard error of three fish. Aasterisks indicate significant (*P* < 0.01) differences. The genes chosen for immune system were: APT2: Antigen peptide transporter 2; CCL4: C-C motif chemokine 4; CD3: T-cell surface glycoprotein CD3 delta chain; CIITA: MHC class II transactivator; CXCL10: C-X-C motif chemokine 10; CXCR4: C-X-C chemokine receptor type 4; FOS: Proto-oncogene c-Fos; IKK1: Inhibitor of nuclear factor kappa-B kinase subunit alpha; IL12: Interleukin-12 subunit beta; IL8: Interleukin-8; IRF3: Interferon regulatory factor 3; LCP2: Lymphocyte cytosolic protein 2; NCF1: Neutrophil cytosol factor 1; NFATC: Nuclear factor of activated T-cells; NFKBIA: NF-kappa-B inhibitor alpha; TLR21: Toll-like receptor 2 type-1; TLR9: Toll-like receptor 9; TNF12: Tumor necrosis factor 12; TNFR11: Tumor necrosis factor receptor superfamily member 11.

We also chose categories including “Carbohydrate Metabolism”, “Energy Metabolism”, “Lipid Metabolism”, “Amino Acid Metabolism”, ‘Metabolism of Other Amino Acids”, “Glycan Biosynthesis and Metabolism”, “Immune System” and “Digestive System” (Table 3) to evaluate metabolic stress information among the three groups. There were 15 pathways related to the immune system, and most genes were over-expressed in the C3 group compared to the C1 and C2 groups (Table 3). In the chemokine signaling pathway, there were 153 and 144 genes up-regulated in the C3 group compared with the C1 and C2 groups. In addition, we chose 20 genes which belonged to immune system to further confirm the results of differentially-expressed analysis using RT-qPCR (Fig. 3).

**Table 3:** The number of genes whose expression changed in the metabolism pathway between two groups.

Pathway		c1-c2 up	c1-c2 down	c1-c3 up	c1-c3 down	c3-c2 up	c3-c2 down
Carbohydrate metabolism							
00562	Inositol phosphate metabolism	3	29	45	11	53	6
00010	Glycolysis/Gluconeogenesis	17	8	52	7	32	8
00500	Starch and sucrose metabolism	10	6	17	19	18	19
00520	Amino sugar and nucleotide sugar metabolism	15	2	31	4	18	11
00620	Pyruvate metabolism	25	3	25	3	18	15
00051	Fructose and mannose metabolism	17	3	37	3	21	8
00052	Galactose metabolism	11	4	29	7	18	7
00640	Propanoate metabolism	19	0	4	1	3	13
00020	Citrate cycle(TCA cycle)	6	4	16	3	13	2
00040	Pentose and glucuronate interconversions	1	1	4	8	7	6
00030	Pentose phosphate pathway	8	7	20	7	11	5
00053	Ascorbate and aldarate metabolism	2	2	0	8	4	6
00630	Glyoxylate and dicarboxylate metabolism	0	2	0	2	2	2
00650	Butanoate metabolism	2	2	4	1	4	2
Energy metabolism							
00190	Oxidative phosphorylation	4	19	25	4	46	0
00680	Methane metabolism	14	8	22	7	9	5
00710	Carbon fixation in photosynthetic organisms	1	5	10	7	8	4
00720	Carbon fixation pathways in prokaryotes	2	0	3	0	2	1
00195	Photosynthesis	0	0	0	0	1	0
00920	Sulfur metabolism	2	0	3	0	2	0
00910	Nitrogen metabolism	4	1	2	0	2	3
Lipid metabolism							
00564	Glycerophospholipid metabolism	15	23	35	32	33	15
00561	Glycerolipid metabolism	13	17	25	26	23	12
00600	Sphingolipid metabolism	7	2	13	6	14	2
00071	Fatty acid degradation	1	3	6	8	7	9
00590	Arachidonic acid metabolism	7	6	17	24	17	12
00565	Ether lipid metabolism	5	4	17	9	16	3
00140	Steroid hormone biosynthesis	4	11	6	18	5	12
0062	Fatty acid elongation	1	5	12	9	12	5
00100	Steroid biosynthesis	14	25	16	8	23	4
01040	Biosynthesis of unsaturated fatty acids	1	9	5	10	6	3
00591	Linoleic acid metabolism	2	4	8	18	6	7
00120	Primary bile acid biosynthesis	2	5	3	9	1	5
00592	alpha-Linolenic acid metabolism	0	5	6	9	8	3
00061	Fatty acid biosynthesis	17	0	1	0	0	1
00072	Synthesis and degradation of ketone bodies	0	1	0	0	3	0
00073	Cutin, suberine and wax biosynthesis	5	0	5	0	3	1
Amino acid metabolism							
00310	Lysine degradation	7	20	19	18	23	11
00330	Arginine and proline metabolism	8	6	16	7	15	7
00270	Cysteine and methionine metabolism	0	7	11	12	10	9
00280	Valine, leucine and isoleucine degradation	0	1	3	2	7	2
00260	Glycine, serine and threonine metabolism	7	11	14	8	7	4
00380	Tryptophan metabolism	4	4	6	1	7	2
00250	Alanine, aspartate and glutamate metabolism	0	4	6	2	5	4
00340	Histidine metabolism	1	5	3	3	7	2
00350	Tyrosine metabolism	0	6	7	3	7	0
00360	Phenylalanine metabolism	1	5	6	3	7	1
00300	Lysine biosynthesis	0	1	0	2	0	0
00290	Valine, leucine and isoleucine biosynthesis	0	0	1	1	0	1
Immune system							
04062	Chemokine signaling pathway	26	13	153	12	144	7
04670	Leukocyte transendothelial migration	25	16	120	18	110	10
04650	Natural killer cell mediated cytotoxicity	25	11	104	9	88	7
04660	T cell receptor signaling pathway	27	17	103	13	88	8
04666	Fc gamma R-mediated phagocytosis	18	19	101	14	92	9
04662	B cell receptor signaling pathway	23	7	93	10	77	8
04620	Toll-like receptor signaling pathway	17	10	76	8	61	7
04640	Hematopoietic cell lineage	23	4	67	7	48	12
04664	Fc epsilon RI signaling pathway	10	15	63	17	58	6
04621	NOD-like receptor signaling pathway	10	4	44	3	39	6
04622	RIG-I-like receptor signaling pathway	7	7	41	7	32	3
04672	Intestinal immune network for IgA production	6	0	25	0	18	0
04612	Antigen processing and presentation	3	3	23	6	22	7
04610	Complement and coagulation cascades	6	2	19	10	20	14
04623	Cytosolic DNA-sensing pathway	4	2	15	2	15	2
Digestive system							
04972	Pancreatic secretion	11	25	65	4	63	6
04971	Gastric acid secretion	8	9	50	4	41	3
04970	Salivary secretion	9	11	42	6	38	5
04974	Protein digestion and absorption	14	20	95	5	91	5
04976	Bile secretion	9	14	31	16	28	9
04973	Carbohydrate digestion and absorption	5	4	25	9	27	7
04975	Fat digestion and absorption	8	8	8	25	4	16
04977	Vitamin digestion and absorption	3	6	1	25	1	6
04978	Mineral absorption	6	3	6	5	2	1

The data in table indicate genes involved in carbohydrate metabolism, energy metabolism, lipid metabolism, amino acid metabolism, immune system function, and digestive system function that were up-regulated and downregulated across groups.

## Discussion

As the cheapest energy sources, carbohydrates are used in fish feeds to improve physical quality and reduce catabolism of proteins and lipids^33^. However, carnivorous fish have a poor ability to utilize high level of carbohydrate. Thus, we investigated the effect of different dietary carbohydrate levels (0%, 18%, and 30%) on growth and health by evaluating the liver transcriptome in *E. akaara*.

In the present study, *E. akaara* fed a diet with 30% carbohydrate content exhibited poor growth performance (PWG = 208.69%) compared with those fed 0% (PWG = 276.16%) and 18% (PWG = 266.26%) carbohydrate content. These results indicated that PWG decreased with an increase in dietary carbohydrate levels. Various mechanisms could account for growth, including endocrine system change^34^. As in mammals, there are two major molecular targets, growth hormone (GH) and insulin-like growth factor (IGF), in the potential endocrine regulation of growth in the teleost^35–38^. They both belong to the GH/IGF-system, which also consists of multiple subtypes of GH receptors (GHRs) and insulin-like growth factor binding protein (IGFBP)^39^.

Several studies have confirmed that IGF and GHR gene expression which is closely related to growth performance can be modulated by the feed component^40–45^. Studies of coho salmon, gilthead sea bream *(Sparus aurata*), and tilapia have indicated that the levels of hepatic GHR and IGF mRNA are positively correlated with body growth rate^46–50^. In accord with these findings, *E. akaara* fed high levels of carbohydrates had the lowest PWG value and the lowest expression of IGF1 and GHR genes (Table 1).

In addition to IGF and GHR, IGFBPs are the main members of the GH/IGF-system. These IGFBPs are traditionally thought to function as carrier proteins and regulate circulating IGF turnover, transport, and distribution. And some studies have shown that IGFBPs are ubiquitously expressed across numerous tissues with autocrine or paracrine effects in salmonids and modulate IGF activities in target tissues^44,51–53^. Except for IGFBP-1, which is predominantly expressed in liver, all other IGFBPs are expressed in many peripheral tissues^45^. In our study, we found that the IGFBP 1, 5, 7 genes were up-regulated in fish fed high levels of carbohydrate diet (C3 group) compared to the other two groups. Elsewise, expression of the IGFBP 2, 3, 4, and 6 genes was reduced in the C3 group, The expression of multiple IGFBPs genes is regulated by different levels of carbohydrate, but little is known about their function in liver. And the mechanism is still not clear. These findings may reinforce the need for additional research about the relationship between IGFBPs gene expression and growth performance in fish.

The liver is an important organ for metabolism and immune function. Significant numbers of natural killer receptor-positive (NKR+) cells and macrophages reside in the healthy liver, which can detect foreign substances and produce inflammatory mediators. In the present study, most genes involved in innate and adaptive immune system were up-regulated following increased dietary carbohydrate levels, suggesting that high levels of carbohydrates can invoke an immune response.

There are many immune system pathways in organism, such as T-cell receptor signaling pathway, B cell receptor signaling pathway, chemokine signaling pathway, Toll-like receptor signaling pathway, RIG-I-like receptor signaling pathway, and NOD-like receptor signaling pathway. In our study, most genes which belongs to immune system pathways were up-regulated in C3 group which fed high carbohydrate level dietary compared with C1 and C2 groups which were fed with lower carbohydrate levels in diet (Table 3). We evaluated 20 genes using RT-qPCR, which belong to 15 pathways in the immune system, and found differential expression across the three groups (Fig. 3). Interleukin 8 (IL8) is a member of the CXC chemokine family and is one of the major mediators of the inflammatory response^54,55^, and, if the harmful antigens are present in the liver, it causes inflammatory-related genes to be over-expressed immediately. In our study, IL8 was more highly expressed in the group fed high levels of carbohydrates compared to the C1 and C2 groups, which had a low constitutive expression (Fig. 3). C-C motif chemokine 4 (CCL4), C-X-C motif chemokine 10 (CXCL10), and C-X-C chemokine receptor type 4 (CXCR4) genes also belong to the chemokine signaling pathway and are chemotactic factors which can active granulocytes, nature kill cells, and monocytes to product interleukin and interferon. In this study, these genes were significantly over-expressed in the group fed high levels of carbohydrates (Fig. 3), indicating the inflammation response in fish^56^.

In the T-cell receptor immune pathway, cluster of differentiation 3 (CD3) induces the T-cell receptor (TCR) to generate an activation signal in T lymphocytes^57^. This high specificity, combined with the presence of CD3 at all stages of T-cell development, makes it a useful immunohistochemical marker for T-cells in tissue sections. T- cells have largely diversified receptors to recognize different antigens, and the MHC class II plays a central role in determining T-cell-mediated adaptive immunity against various pathogens. In this study, MHC class II and CD3 genes had the highest expression in the group fed high levels of carbohydrates compared to the groups fed few and moderate levels. These results indicate that high levels of carbohydrates induced an adaptive immunity response.

Toll-like receptors (TLRs), inhibitor of nuclear factor kappa-B kinase subunit alpha (IKKA), caspase-8, proto-oncogene c-Fos (Fos), interferon regulatory factor 3 (IRF3), tumor necrosis factor (TNF), and tumor necrosis factor receptor (TNFR) belong to Toll-like receptor signaling pathway, NOD-like receptor signaling pathway, and RIG-I-like receptor signaling pathway. These three pathways are cytoplasmic pattern recognition receptors (PRRs), which are involved in the recognition of viruses by the innate immune system^58^. PRRs responded to many exogenous pathogens and/or endogenous serious signals, by recognizing highly conserved structures, such as pathogen associated molecular patterns (PAMPs) and danger/damage associated molecular patterns (DAMPs). Following recognition by PRRs, downstream signaling pathways induce expression of genes of inflammatory molecules that can activate the cytokine signaling pathway to regulate innate and adaptive immunity^59^. The genes belonging to these three pathways had significantly higher expression in the group fed high levels of carbohydrates, suggesting that an imbalance in carbohydrates signals the liver to produce immunocompetence through the innate and adaptive immune system.

In Atlantic salmon, another sea carnivorous fish, numerous studies reported plant feedstuffs could lead to the changes of histomorphological and immune response related genes expression in intestine at least four weeks^60–65^. However, Sahlmann *et al*.^66^ found that the gene expression changes could be detected in third day, which is earlier than signs of inflammation in histological evaluation. In this study, we got the liver transcriptome data of *E. akaara* after 8 weeks of rearing experiment and it showed that the fish consuming high level of carbohydrate may increase inflammation response. Then, we did a short feeding trial with C2 and C3 experimental diets to make up the defect of functional verification in original experiment. After 2 weeks, although there was no difference in histological section of liver tissue, the immune response related genes were higher expression in C3 group than C2 group by RT-qPCR. It suggested that the high level of carbohydrate had the tendency to give rise to immune reaction in *E. akaara*. On the other hand, we used 3 fishes of each replicating group per treatment as the RNA templet to confirmed the sequencing data with RT-qPCR. They were consistent with each other. These results all confirmed that our data and conclusion is credible.

## Conclusion

The liver transcriptome data of *E. akaara* reported here indicate that high carbohydrate level of diet can lead to poor growth and inflammatory immune response in *E. akaara*. The up-regulation of a large number of genes in immune system pathway revealed the fish maybe experience inflammatory response which may be related with a decrease in growth rate. The prominent effects caused by diet composition on immune function parameters underline the importance of nutritional factors in the defense system of *E. akaara*.

## Materials and Methods

### Experimental design and sample preparation

This study was implemented at the Zhejiang province Key Lab of Mariculture and Enhancement in Zhoushan, China. Before the experiments, the juvenile fish were temporarily fed a commercial diet. At the beginning of the experiment, fish (initial weight 7.79 ± 0.01 g) were weighed and sorted into 9 cages (20 fish/cage). The experimental system consisted of 9 net cages (60 × 60 × 80 cm; L × W × H). All net cages were placed in one large concrete tank (13.0 × 4.0 × 1.5 m). A recirculating water system (including a sedimentation chamber, one drum filter, one fluidized sand filter, two biofilters, and two protein skimmers) was used during the experimental period. Three replicate groups of fish were used for each experiment diet. Three semi-purified isoproteic (48%) and isolipidic (9%) diets were formulated with different levels of corn starch (C1: 0%; C2: 18%; C3: 30%). All fish were fed to apparent satiation twice daily at 8:30 and 16:00, and the feeding trial lasted for 8 weeks.

During the experimental period, dissolved oxygen (DO), salinity, and temperature were 6.2 ± 0.1 mg/L, 25.1 ± 0.9 g/L and 27.5 ± 2.3 °C, respectively. At the end of the feeding trial, three fish in each cage were dissected, livers were immediately frozen in liquid nitrogen, and then they were stored at −80 °C until RNA extraction for sequencing. The experiment was implemented at the Zhejiang province Key Lab of Mariculture and Enhancement of Zhejiang Ocean University (Zhoushan, China). All experimental procedures were approved by the Local Animal Care and Use Committee (Zhejiang, China), and the study was carried out in accordance with regulations for the administration of affairs concerning experimental animals of China (Promulgated by Decree No. 2 of the State Science and Technology Commission on November 14, 1988).

### RNA extraction and sequencing

Total RNA was extracted from livers of each group by homogenization in 1 ml TRIZol (Invitrogen) following the manufacturer’s instructions. The concentration and quality of total RNA were determined by electrophoresis (Thermo Scientific Nano drop 2000, USA). And total RNA was used for paired-end RNA sequencing. Library construction and sequencing were performed on an Illumina HiSeq. 2000 sequencer according to manufacturer’s specifications.

### De novo assembly and functional annotation

Raw reads were pre-processed by discarding reads with adaptors, with unknown sequence (N) proportion greater than 5%, and those of low quality (Phred quality score <30). Then, raw reads were assembled by Trinity software using default parameters^67^. Transcripts shorter than 200 bp were removed from the subsequent analysis^68^. The assembly sequencing results evaluated used bowtie-1.0.1^69^, which mapped the raw reads to the assembled transcriptome. Transcripts from the previous step were translated in all six possible open reading frames (ORFs) with TransDecoder tool, which was used to predict the ORF of the RNA sequencing assembly sequence. Proper translation was defined as one that gave the longest amino acid sequence^70^ according to the hidden Markov model (HMM).

The functional annotations of predicted amino acid sequences were performed using Trinotate (http://trinotate.sourceforge.net/) through searching against the Uniprot Knowledgebase and Swiss-Prot. In addition, we ran HMMER, signalIP, and tmHMM in Trinotate to identify protein domains, predict signal peptides, and transmembrane regions, respectively.

Gene Ontology (GO) annotations of transcripts were obtained by searching against the non-redundant (nr) database using the Blast2GO program^71^ with an E-value cutoff of 10^−5^. GO functional classifications were performed using WEGO software^72^. Kyoto Encyclopedia of Genes and Genomes (KEGG) pathways were assigned to the assembled transcripts using the online KEGG Automatic Annotation Server (KAAS) (http://www.genome.jp/kegg/kaas/). The bi-directional best-hit (BBH) method was used to obtain KEGG orthology assignments.

### Identification of differentially-expressed genes (DEGs) and RT-qPCR

DEGseq^73^ was used to compare RNA-seq data and identify differentially expressed genes. And the input of DEGseq is uniquely mapped reads from RNA-seq data with a gene annotation of the corresponding transcript expression values provided by RPKM^74^. A set of 20 genes which were significantly affected by high carbohydrate food were quantified by RT-qPCR to validate sequencing performance. Primers were designed using Primer premier 6 software (Table 4). The *β*-actin as the reference gene was quantified. For RT-qPCR, 2 μg of total RNA per sample was reverse transcribed into cDNA using the PrimeScript™ II 1st Strand cDNA Synthesis Kit (Takara), following manufacturer’s instructions. Amplifications were carried out in triplicate in a final volume of 20 μl containing 2 μl cDNA (1/10 dilution), 0.5 μM of each primer and 10 μl SYBR Green Supermix (Takara). The RT-qPCR profiles contained an initial activation step at 95 °C for 10 min, followed by 40 cycles: 15 s at 95 °C, 15 s at the specific primer pair annealing temperature (Ta; Table 4) and 15 s at 72 °C. After the amplification phase, a melt curve of 0.5 °C increments from 75 °C to 90 °C was performed, enabling confirmation of the amplification of a single product in each reaction. The analysis was based on the Ct values of the PCR products. Results are shown as changes in relative expression normalized with β-actin using the 2^−ΔΔCt^ method. Chi-square statistic was applied to detect significant differences in each group.

**Table 4:** Primers used for RT-qPCR analyses.

Gene name	Primer sequence (5′–3′)	Fragment (bp)	Ta (°C)
TAP2	TGGTTCGCAGCACAGTCA	262	56
	GCCTCGTTATACCTCCTCAGT		
Caspase 10	GCTACGGACAAGACATAC	257	55
	GGTGGATGATGAGGAGAA		
CCL4	TCTCGCTCTGTCTGTGTT	251	56
	CGTCCAGGTAGGTGATGA		
CD3	TTCCAGTACCACAAGACAG	159	53
	CCAGGACTCAGAGGTGTA		
CIITA	GTCGGTTAGTCTGCTTGGT	359	55
	TGTTCTGTCTGCTTCCTCTC		
CXCL10	TCTACCAAGCGACCATCT	290	54
	GTGTCAGTGCTGTCAGTATA		
CXCR4	GACTCGGACTCTGTTGAC	273	54
	CTGTGTTGGCATCTTCTTG		
FOS	GGAGGTTGAGGTGTAGGT	216	56
	GCTAATCTGTTGAAGGAGAAG		
IKK1	TTAACTCTTCTGGAACCTCTC	263	55
	GTGACCTGACCGTATGGA		
IL12	CCTCACCATCTACATACACAT	190	55
	CACCTTGACCTGGAACTG		
IL8	CCATCTGAGGAGAAGAACTC	381	56
	CTGTGTTATTGAGCCTGATTC		
IRF3	GGAGTCGGCTTGAAGATA	246	55
	TCAGTGTGGAAGAGGAAC		
LCP2	AGTCTGTCTGGCTGTGAT	151	53
	TCTCCTCCTTCTTGCTGAT		
NCF1	TGGTGGTTCTGTCAGTGT	376	58
	CCTTGTTGTGGATGCTCTT		
NFATC	GTCTGCTTCATACTCGTCTAT	136	53
	TGTCTGCCACTCTGTCAA		
NFKBIA	TGTTGAGGAAGTCTGTGTT	230	56
	GCTGAAGGAGGAGGAGTA		
TLR2	ACTCTGGAGGTGTTGGAT	280	56
	CGTTGATGGCTGATTGGA		
TLR9	CCTACCTTGACATCTCTGAC	282	52
	CCGCCTTACTGAAGAACAT		
TNF12	CTCGCCTCACATCTTCAG	220	52
	TCCACAACAACTTCCACAA		
TNFR11	CAGTGCTGTCAGTCATCA	127	52
	TGTATCCTCGTCCTCTTCA		
β actin	CGACCTCACAGACTACCT	221	56
	AACGGAACCTCTCATTGC		

### Growth performance calculations

Data on initial body weight were used to calculate percentage weight gain (PWG). PWG (%) = 100% × (final body weight − initial body weight)/initial body weight. All measured values were presented as the mean ± standard deviation (SD). All data were tested for homogeneity (Levene’s test) and normal distribution (Kolmogorov-Smirnov tests), and necessary data were transformed before analysis. When data did not present variance homogeneity and normal distribution, Spearman’s correlation was used to determine the relationship between the response data and dietary carbohydrate. Values were regarded as statistically significant at *P* < 0.05. Statistical analysis was used to rank the groups using SPSS 18.0 (IBM, Chicago, USA) for Windows.
